# Development and pre-clinical evaluation of a Zika virus diagnostic for low resource settings

**DOI:** 10.3389/fmicb.2023.1214148

**Published:** 2023-11-20

**Authors:** Rickyle Balea, Nina M. Pollak, Jody Hobson-Peters, Joanne Macdonald, David J. McMillan

**Affiliations:** ^1^Centre for Bioinnovation, University of the Sunshine Coast, Sippy Downs, QLD, Australia; ^2^School of Science, Technology and Engineering, University of the Sunshine Coast, Sippy Downs, QLD, Australia; ^3^School of Chemistry and Molecular Biosciences, The University of Queensland, St Lucia, QLD, Australia; ^4^Australian Infectious Diseases Research Centre, The University of Queensland, St Lucia, QLD, Australia; ^5^BioCifer Pty Ltd., Auchenflower, QLD, Australia

**Keywords:** Zika virus, NS1 gene, recombinase aided amplification, lateral flow detection, point-of-care, rapid test

## Abstract

**Introduction:**

Zika virus (ZIKV) is a re-emerging flavivirus that poses a significant public health threat. ZIKV exhibits a wide array of non-vector borne human transmission routes, such as sexual transmission, transplacental transmission and blood transfusion. Detection and surveillance of ZIKV is considered paramount in prevention of major outbreaks. With the majority of cases reported in low-resource locations, simple, low-cost detection methods are considered highly desirable.

**Materials and Methods:**

Here we have developed a sensitive and specific ZIKV diagnostic using reverse transcription recombinase-aided amplification (RT-RAA) coupled with lateral flow detection (LFD) targeting a highly conserved region of the ZIKV NS1 gene.

**Results:**

We show our rapid, isothermal-ZIKV-diagnostic (Iso-ZIKV-Dx) can detect 500 copies of synthetic ZIKV RNA/μL in under 30 min at a constant 39°C. Using simulated urine samples, we observed that Iso-ZIKV-Dx also detects as low as 34.28 RNA copies/reaction of ZIKV (MR766 strain). Specificity testing confirmed that our test does not detect any co-circulating flaviviruses (dengue, West Nile, Japanese encephalitis, Murray Valley encephalitis and yellow fever viruses) or chikungunya virus. Sample processing results show complete inactivation of ZIKV (MR766 strain) in 5 min at room temperature using our novel viral RNA sample preparation reagent. Furthermore, lateral flow strips testing demonstrates positive diagnoses in as little as 5 min in running buffer.

**Discussion:**

Contrary to conventional RT-qPCR, our Iso-ZIKV-Dx does not require expensive machinery, specialised laboratory settings or extensively trained personnel. Pre-clinical evaluation demonstrates that our test exhibits robust, in-field capabilities without compromising sensitivity or specificity. When compared to the gold-standard RT-qPCR, our Iso-ZIKV-Dx test offers an array of applications that extend beyond diagnostics alone, including potential for surveillance and monitoring of ZIKV vector competency.

## GRAPHICAL ABSTRACT

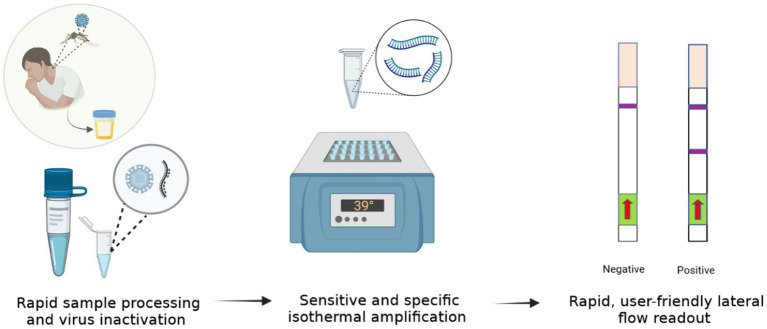

## Highlights

- Development of a low-resource rapid Zika virus diagnostic test.
- Rapid one-step sample processing protocol inactivating Zika virus in 5 min.
- Test format utilising isothermal amplification coupled with lateral flow detection.
- Achieved detection of highly virulent ZIKV MR766 in under 30 min.
- Rapid Zika virus test 4 times faster than RT-qPCR.

## Introduction

Zika virus (ZIKV), a member of the *Flaviviridae* family, was first isolated in 1947 from the serum of a rhesus monkey in Uganda (Dick et al., 1952). In 2007, an outbreak on Yap Island (Hayes, 2009) resulting in the first instance of ZIKV transmission outside of Africa and Asia was reported. ZIKV outbreaks have subsequently been reported in multiple locations including French Polynesia (Roth et al., 2014), Papua New Guinea (Chang et al., 2016), New Caledonia (Dupont-Rouzeyrol et al., 2015), and Brazil (Wen et al., 2017). Spanning throughout nearly all seven continents, ZIKV remains a priority disease by World Health Organisation (WHO) (TwistDx^™^, 2023b). Although predominantly transmitted via mosquitos (*Aedes albopictus* and *aegypti*) (Azar and Weaver, 2019), blood transfusion and sexual transmission have also been reported (Gregory et al., 2017). Historically ZIKV transmission was primarily observed in remote, developing countries (Paixão et al., 2016). However, in 2019, the first locally acquired cases of ZIKV transmission were reported in southern Europe (Brady and Hay, 2019), initiating the prevalence and spread of ZIKV in developed nations. Most ZIKV infections manifest in mild, flu like symptoms (Mumtaz et al., 2016). In severe cases, medical complications such as Guillain Barre (Hendel-Paterson et al., 2016) and severe thrombocytopenia (Sharp et al., 2016) have been reported. ZIKV infection mechanisms also allow for cross placental infections, resulting in microcephaly in developmental infants (Wen et al., 2017).

With no approved vaccine or therapeutics (Da Silva et al., 2018), rapid and accurate detection of ZIKV is a crucial component in predicting and monitoring potential outbreaks (Heukelbach et al., 2016). The majority of ZIKV-positive individuals, including pregnant women are also asymptomatic (Paixao et al., 2018). Crucially delayed diagnosis among pregnant women is particularly concerning due to the inherent risk of congenital abnormalities. Current ZIKV detection and diagnostic strategies utilise both nucleic acid amplification tests (NAATs) (Gourinat et al., 2015) or antibody-based detection-based techniques (Kadkhoda et al., 2017). Due to antigenic cross-reactivity between Zika antigens and other flaviviruses (Stettler et al., 2016), serology based testing such as Enzyme Linked Immunosorbent Assay (ELISA) are less favoured. Reverse Transcription-Quantitative Polymerase Chain Reaction (RT-qPCR) therefore, still remains the ‘gold standard’ for detection and diagnostics among arboviruses (Dong et al., 2012). While accurate and sensitive, a draw-back for RT-qPCR is the need for specialised equipment and trained personnel that restricts these tests to centralised laboratories.

A diagnostic platform that enables in-field or point-of-care (POC) detection without the need for highly trained personnel or specialised equipment offers beneficial attributes to both clinical diagnostics and surveillance of not just ZIKV, but arboviruses alike (Ahmed et al., 2022). Isothermal NAAT’s address all these issues and have been described as potential alternatives to RT-qPCR for viruses such as Zika (Cheikh Tidiane Diagne et al., 2020). As of recent years, innovation and proliferation of various isothermal amplification tests have taken significant strides within the field of rapid diagnostics (Xue et al., 2020). Recombinase aided amplification (RAA) (Jiangsu Qitian Gene Biotechnology Co., Ltd, 2023) is a promising isothermal technique that utilises similar molecular mechanisms as Recombinase Polymerase Amplification (RPA) (TwistDx^™^, 2023a). For RNA viruses such as ZIKV, a reverse transcriptase enzyme is added to the RAA reaction. Comparable to RT-qPCR, reverse transcriptase-recombinase aided amplification (RT-RAA) has been shown to be both rapid (Xue et al., 2020) and clinically sensitive (Wang et al., 2020). However, NAAT test uptake for low-resource detection of disease is hampered by the lack of field-friendly sample preparation techniques, instead requiring purification of RNA using magnetic beads or column-based laboratory technologies (Pollak et al., 2022).

Here we describe a rapid, isothermal ZIKV diagnostic (Iso-ZIKV-Dx). Our test format combines a unique low-resource sample preparation reagent with RT-RAA amplification, and lateral flow detection (LFD) to enable ZIKV detection in urine in under 30 min without the need for expensive nor advanced instrumentation. As such, our Iso-ZIKV-Dx exhibits promising diagnostic capabilities suitable for low-resource settings.

## Materials and methods

### Plasmids and RNA template preparation

Plasmids (pBIC-A) containing regions of the ZIKV Envelope (E) gene fragment (OM964568.1, 901–2,412 nt) and non-structural 1 (NS1) gene fragment (MW015936, 249–3,545 nt) of ZIKV were obtained from Bioneer Pacific Pty Ltd., Victoria, AUS. The pBIC-A- NS1 and E gene vector was transformed into *E. coli*. A single colony of *E. coli* containing the pBIC-A- NS1 plasmid was streaked onto LB broth agar supplemented with 100 μg/mL of ampicillin and incubated at 37°C. The selected colony was grown in liquid LB broth supplemented with 100 μg/mL of ampicillin for plasmid isolation using the ‘NucleoBond Xtra Midi’ kit (Machery-Nagel, GER). MEGAscript (Ambion, Austin USA) *in vitro* transcription kit was used to yield RNA transcript from pBIC-A- NS1 plasmids. XhoI was the chosen restriction enzyme to digest the linearize plasmid in preparation for *in vitro* transcription. Transcribed RNA from pBIC-A- NS1 plasmid was quantified using Quibit RNA Hs Kit. Using software, a calculation of an approximate numerical quantity of RNA copies/μL from extracted RNA was established (Pollak et al., 2023c). RNA transcripts were then stored at −80°C as working stocks and used as reference RNA standards for qRT-PCR.

### RAA primer and probe design

**Figure 1:**
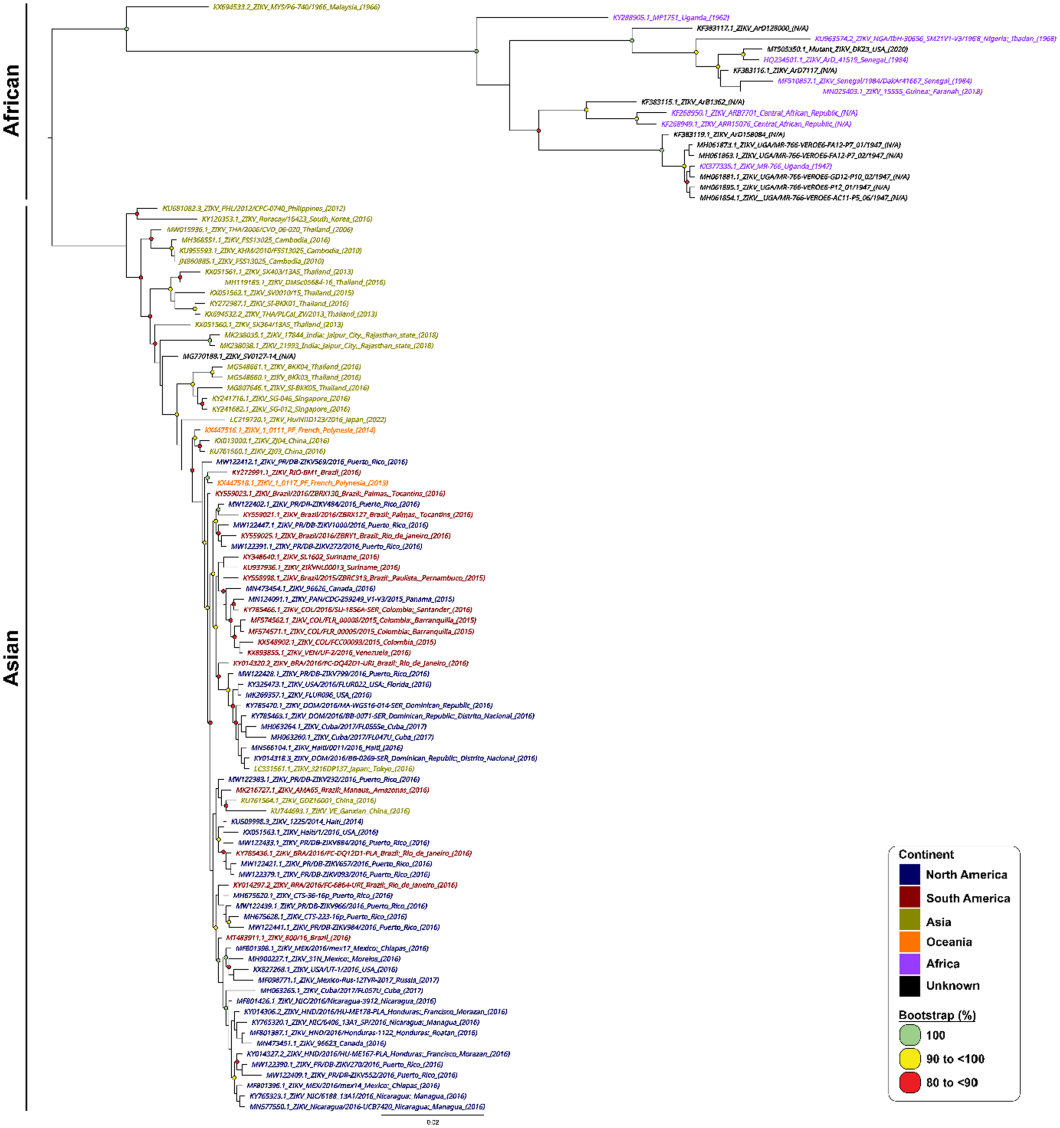
Phylogenetic tree of 105 unique ZIKV NS1 gene sequences. Phylogenetic relationships among 105 ZIKV NS1 sequences. ZIKV NS1 sequences are named by their corresponding GenBank accession number/strain/country of origin, with text being coloured according to their continent of isolation. The midpoint-rooted maximum likelihood phylogenetic trees were constructed using IQTREE2, which automatically incorporates the most appropriate nucleotide substitution model (TN + F + G4). The scale bar indicates the number of nucleotide substitutions per site. Bootstrap values above 80% are displayed next to the node of each clade and are coloured according to the figure legend.

A total of 967 ZIKV NS1 genes obtained from NCBI’s databank representing both the African and Asian were aligned using Geneious Prime (version 2023.0.4) (Kearse et al., 2012). The first phylogenetic tree was created to isolate only unique ZIKV NS1 sequences. A total of 105 unique sequences were isolated and then imported to Geneious Prime for re-alignment using MAFFT (Nakamura et al., 2018). To assess the range of ZIKV sequences targeted by the primers and probes, a maximum likelihood (ML) phylogenetic tree (Figure 1) was constructed using IQTREE2 (Minh et al., 2020), utilising 10,000 bootstrap replicates and the TEST function. A consensus sequence of all unique ZIKV NS1 gene sequences was created using BioEdit 7.2 (Informer Technologies, INC, GER). Primers and probes were then designed using the most conserved region of the NS1 gene within the consensus sequence. Primer and probe design were further evaluated (primer dimers, secondary structures and GC content) using an oligoevaluator (Sigma-Aldrich Co. LLC, 2014). ALL primers and probes were bioinformatically evaluated to ensure no non-specific annealing to other flavivirus NS1 sequences occurred. Probes and primers (Table 1) were synthesised by (Bioneer Pacific Pty Ltd., Victoria, AUS) and purified via PAGE and HPLC, respectively.

**Table 1:** Primer and probe sequences for rapid Iso-ZIKV-Dx test.

Oligonucleotide	Sequence
Forward primer	GAAATYCGGTTTGAGGAATGYCCAGGHACYAAGG
Reverse primer	[5’ Biotin]GGTTCYTTYCTGGGCCTTATCTCCATTCCATACC
Probe	[5’FAM]GAGGACCATCTCTGAGATCAACYACTGCAAG[Internal dS Spacer]GGAAGGGTSATHGAG [3’ C3 spacer]

### Iso-ZIKV-Dx test

#### Rapid sample processing

ZIKV MR766 culture was mixed into urine and RPMI media samples at a ratio of 1:1 with TNA-Cifer Reagent E (BioCifer, Auchenflower, AUS) at room temperature for 5 min. Urine and RPMI media samples were diluted 1:5 in nuclease free H_2_0.

#### RT-RAA amplification

Each RT-RAA test was performed using the RAA kit (Jiangsu Qitian Gene Biotechnology Co. Ltd., Wuxi City China) with final reaction conditions of 0.83x RAA rehydration buffer, 1/5 RAA pellet, forward primer (350 nM), reverse primer (350 nM), probe (200 nM), Endonuclease IV (2U; New England Biolabs, Victoria, AUS), Moloney Murine Leukemia virus reverse transcriptase enzyme (M-MLV, 60U; Biocifer Pty Ltd., Auchenflower, AUS), magnesium acetate (MgOAc, 23.33 mM) and 2 μL template in a final reaction volume of 12 μL. MgOAc was added to the cap of each 0.2 mL PCR tube. Once template had been added, tubes were centrifuged and incubated at 39°C for 20 min using a heating block.

#### Lateral flow strip detection

HybriDetect lateral flow strips (LFS) (Milenia Biotec, Giessen, GER) were treated with 8 μL of 0.4% casein blocking buffer for pre-activation (Rames and Macdonald, 2019). To each strip, 2 μL of amplicon was pipetted on to the sample pad. The LFS strips were placed into 2 mL Eppendorf tubes containing 100 μL of LFS Running Buffer (Li et al., 2019) for 5 min. LFS were scanned using an Epson Perfection V39 Flatbed Scanner (Epson, New South Wales, AUS). The scanned images were converted to greyscale using Irfan View 64 and then imported to ImageJ for analysis. Band intensity analysis and statistical quantification were conducted as previously described (Pollak et al., 2023b).

#### Sensitivity and specificity testing

Analytical sensitivity testing for Iso-ZIKV-Dx tests were performed using a 10-fold serial dilution of purified, synthetic RNA transcripts coding for ZIKV E and NS1 gene fragments. Analytical specificity testing utilised purified RNA of various virus strains (Table 2). Synthetic ZIKV RNA transcript (1 × 10^6^ copies/μL) was used as a positive control for validation of specificity.

**Table 2:** Virus strains used in this study.

Virus	Abbreviation	Strain	GenBank accession number
Zika virus	ZIKV	MR766	KX830960
Chikungunya virus	CHIKV	Mauritius 2006	MH229986
Dengue virus serotype 1	DENV-1	ET00.243	JN415499
Dengue virus serotype 2	DENV-2	ET00.300	JN568254
Dengue virus serotype 3	DENV-3	East Timor 2000	JN575585
Dengue virus serotype 4	DENV-4	ET00.288	JN571853
Japanese encephalitis virus	JEV	Nakayama	EF571853
Murray Valley encephalitis virus	MVEV	1–51	AF161266
West Nile virus	WNV_KUNV_	Kunjin strain NSW 2011	JN887352
Yellow fever virus	YFV	17D	MT505351

#### Simulated-infected urine

ZIKV culture 3.56 × 10^8^ TCID_50_/ml was spiked into Pickering Laboratories #1700–0018 Artificial urine medium for growing urological pathogens (Walker Scientific Pty Ltd., Joondalup DC, AUS).

### Viruses and cell culture

All viral strains used in this study are listed in Table 2. Excluding ZIKV, all viruses were obtained from Hobson-Peters lab (University of Queensland, St Lucia, QLD) and were cultured at high titres as previously described (Hobson-Peters et al., 2013).

#### Cell culture

*Aedes albopictus* larvae cells C6/36 (ATCC-CRL-1660™) were obtained from the ATCC. Cell lines were cultured in Gibco (USA) 1640 RPMI supplemented with 5% Foetal Bovine Serum (FBS), 2 mM L-glutamine, Gibco (USA) 1x Antibiotic/Antimycotic at 28°C in a 5% C0_2_ incubator until approximately 80% confluent (Pollak et al., 2023b).

### ZIKV infection and titre determination

#### Virus culture

ZIKV MR766 culture (accession MK105975) was used to infect C6/36 cells at a multiplicity of Infection (MOI) of 0.01 for 5 and 7 days, respectively (Pollak et al., 2023a). Virus culture media was harvested after centrifugation at 4°C for 10 min at 130 × *g* and then stored at −80°C.

#### Titre determination

ZIKV titre determination was evaluated by standard TCID_50_ assays and fixed-cell ELISAs using C6/36 cells in a 96 well plate as previously described (Pollak et al., 2023b). Titres were calculated using the Reed and Muench method (Reed and Muench, 1938).

#### ZIKV inactivation testing

ZIKV inactivation testing was performed using ZIKV culture (3.56 × 10^8^ TCID_50_/ml) mixed with TNA-Cifer Reagent E (TCE; BioCifer, Auchenflower, AUS) at different ratios (1:1 and 2:1, ZIKV culture to TCE) and incubated from 0 min up to 10 min. Titre was determined as previously described (Pollak et al., 2023b).

### RNA purification

RNA from viral stocks were purified using TRIzol™ (Invitrogen by Thermo Fisher Scientific Pty Ltd., Victoria, AUS) or column-based kit (NucleoSpin RNA Virus Isolation Mini kit, Machery-Nagel, Duren, GER) following protocols outlined by the manufacturer. Viral RNA was eluted into 150 μL of nuclease free H_2_O and stored at −80°C.

### ZIKV RT-qPCR

For a ZIKV RT-qPCR comparative, TaqMan™ Fast Virus 1-Step Master Mix (Thermo Fisher Pty Ltd., Victoria, AUS) was used in conjunction with primers and probes previously described (de Moraes et al., 2018). Parameters for RT-qPCR were implemented as per manufacturer’s instructions.

## Results

In designing our primers and probes, we performed stringent bioinformatic analysis to ensure our primers and probes target as many strains and isolates of ZIKV from all existing lineages as possible. We mapped out all known ZIKV sequences from NCBI covering both Asian and African lineages of ZIKV and constructed a phylogenetic tree using the most unique 105 sequences (Figure 1).

### Analytical sensitivity

**Figure 2:**
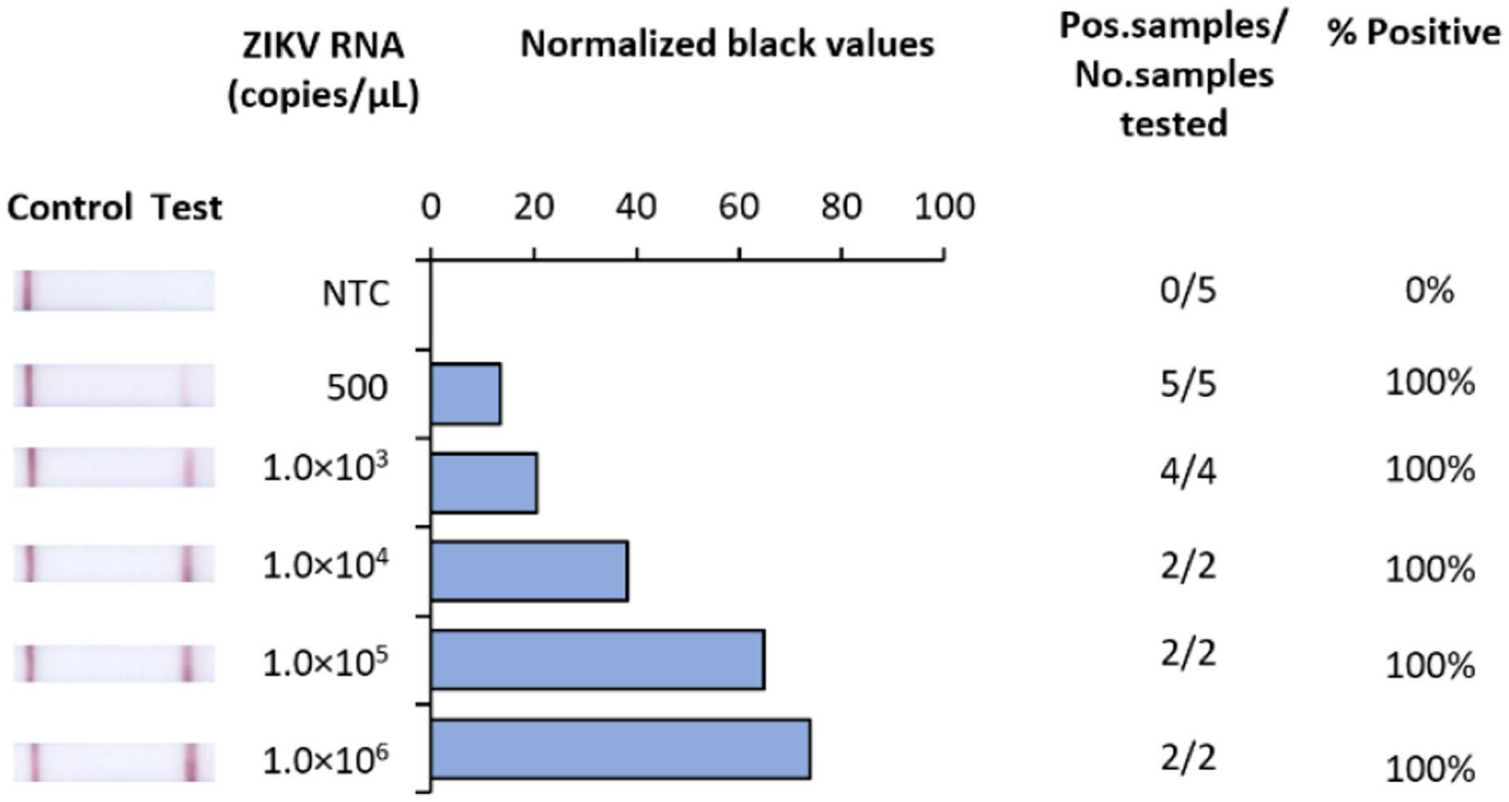
Analytical sensitivity of Iso-ZIKV-Dx using synthetic RNA transcripts. Sensitivity tests used 10-fold serially diluted synthetic ZIKV RNA. Scanned images of lateral flow strips with two lines (control and test) indicating positive detection of ZIKV and one being negative; no template control (NTC) consisted of nuclease free H_2_O in place of RNA (left). Normalised pixel density (black shifted) contrasted with the white space on lateral flow strips (middle). Number of positive samples detected over number of samples tested along with calculated percentage accuracy of positive results for each dilution series (right).

In developing a rapid, low-resource reliant diagnostic for ZIKV, we strategically designed RT-RAA primers and probes to target a highly conserved region of the NS1 gene. To test analytical sensitivity, serial dilutions of RNA transcripts were assessed. Our RT-RAA tests exhibited a limit of detection (LOD) of 500 RNA copies/μL (Figure 2).

### Analytical specificity

**Figure 3:**
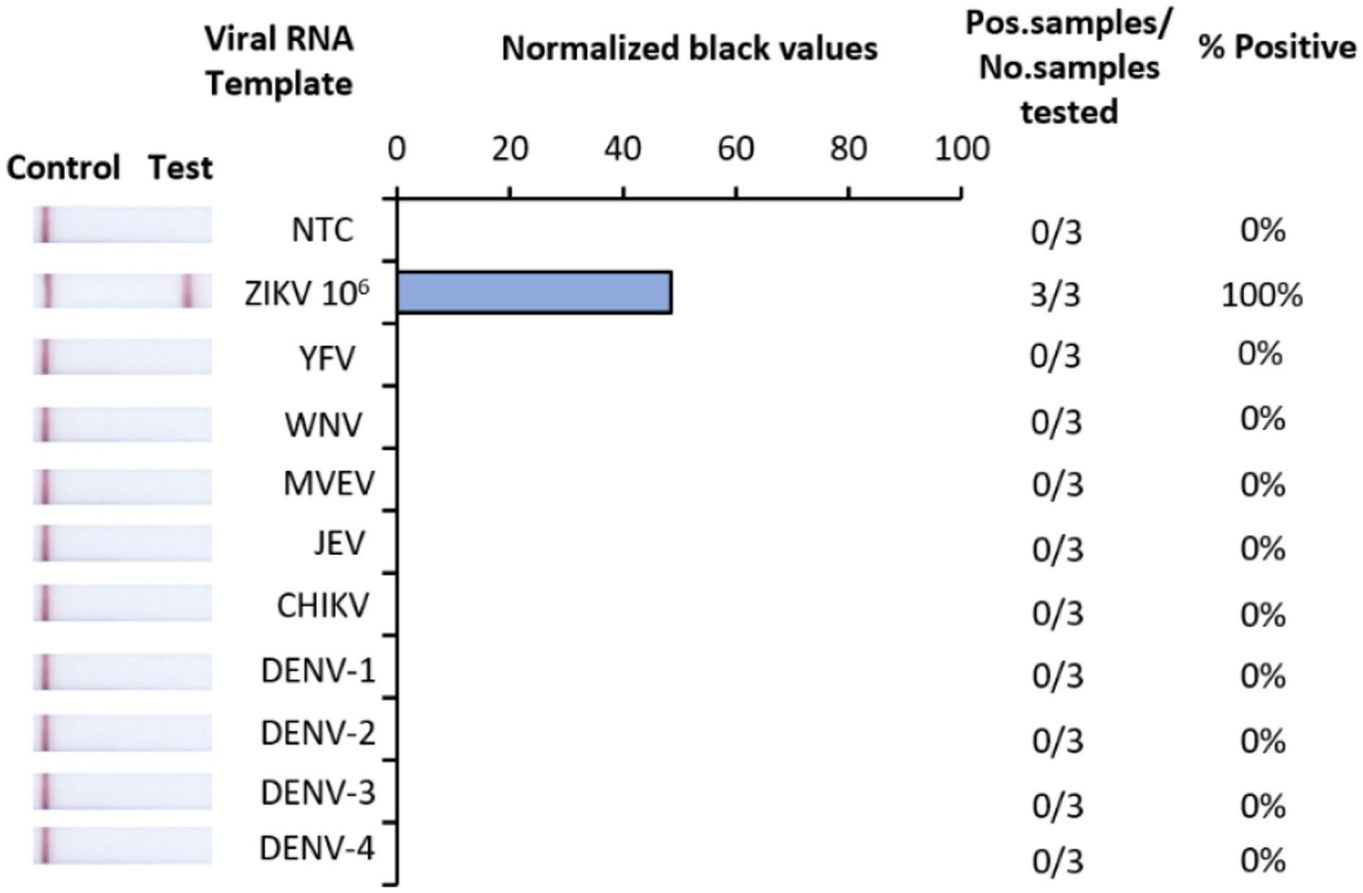
Analytical specificity of Iso-ZIKV-Dx using co-circulating flaviviruses and chikungunya virus. Specificity tests used synthetic RNA from ZIKV (PTC) and TRIzol extracted RNA from yellow fever virus (YFV), West Nile_Kunjin_ virus (WNV_KUN_), Murray Valley Encephalitis virus (MVEV), Japanese Encephalitis virus (JEV), chikungunya virus (CHIKV), dengue 1, 2, 3 and 4 virus (DENV-1, 2, 3, and 4). Scanned images of lateral flow strips with two lines (control and test) indicating positive detection of ZIKV and one being negative; no template control (NTC) consisted of nuclease free H_2_O in place of RNA (left). Normalised pixel density (black shifted) contrasted with the white space on lateral flow strips (middle). Number of positive samples detected over number of samples tested along with calculated percentage accuracy of positive results (right).

The analytical specificity of Iso-ZIKV-Dx was assessed against RNA from eight common flaviviruses that co-circulate with ZIKV (Table 2). Alphavirus chikungunya virus (CHIKV) (Table 2) was also included due to its common co-circulation with ZIKV. Our results showed that none of the common co-circulating viruses were detected using Iso-ZIKV-Dx (Figure 3). In addition, bioinformatic analysis of different ZIKA isolates indicated that the designed primers and probes are homologous to all ZIKV strains (African and Asian lineages) (Supplementary Figure 1).

### Sample preparation inactivates ZIKV

**Figure 4:**
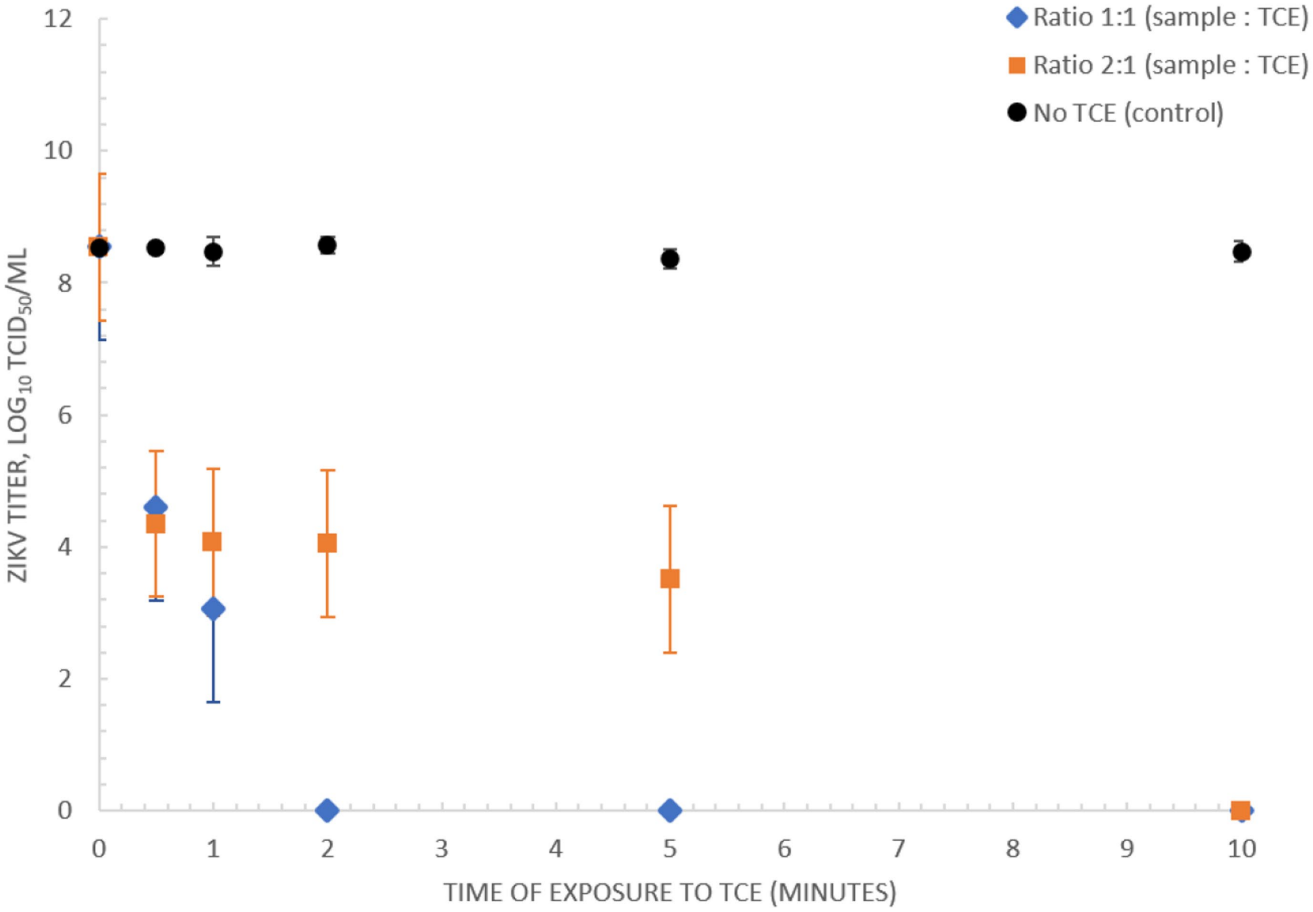
Inactivation of ZIKV (MR766) culture using TNA-Cifer Reagent E. Inactivation of 3.56 × 10^8^ TCID_50_/mL ZIKV (MR766) culture using TNA-Cifer Reagent E (TCE) at 1:1 and 2:1 ratio (sample to TCE) incubated for 0, 0.5, 1, 2, 5, and 10 min at room temperature.

A rapid diagnostic suitable for low-resource implementation requires a simple sample preparation procedure. Here we assessed the capacity of TNA-Cifer Reagent E (TCE) (BioCifer, Auchenflower, QLD, AUS) as a sample preparation reagent for rapid inactivation and sample preparation of ZIKV. Our results showed that ZIKV MR766 (3.56 × 10^8^ TCID_50_/mL) was completely inactivated after 5 min when using a 1:1 ratio of sample to TCE, and 10 min when using a 2:1 ratio (Figure 4).

### Detection of ZIKV in synthetic urine and RPMI medium

**Figure 5:**
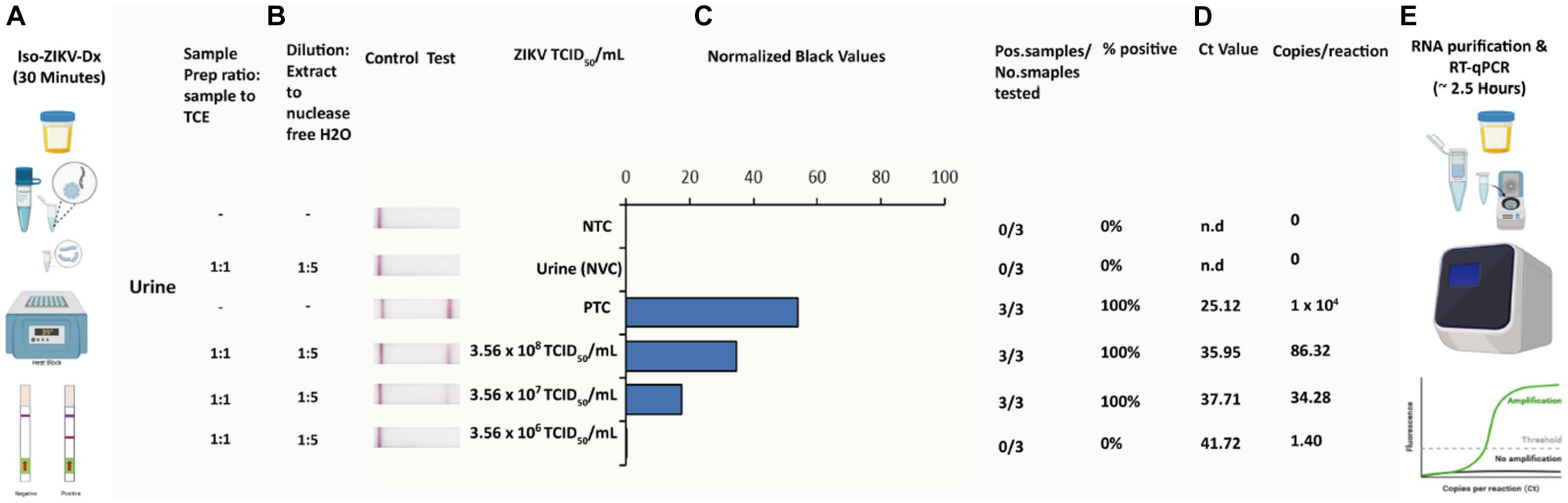
Rapid Iso-ZIKV-Dx of simulatedurine samples spiked with ZIKV (MR766). **(A)** Workflow, equipment needed and time frame of Iso-ZIKV-Dx. **(B)** Sample processing conditions including sample to reagent ratio and processed sample dilution ratio. **(C)** Sample description and quantities (NTC, non-template control; PTC, positive template control, synthetic ZIKV RNA transcripts 10^6^ copies/μl); NVC, no virus control, urine. Scanned lateral flow strips showing test and control bands observable by naked eye. Normalised pixel densities (black values) from the displayed lateral flow strips. **(D)** Comparative Ct values and copies/reaction quantified via TaqMan RT-qPCR. **(E)** Workflow, equipment and time frame involved in conventional ZIKV RT-qPCR diagnostic.

A key principle of our rapid Iso-ZIKV-Dx is operation in a manner that is not dependant on intricate resources or involve complex methodology. As such, the use of urine as a clinical matrix (Gourinat et al., 2015; Bonaldo et al., 2016; Lamb et al., 2016) eliminates the requirement for excessive sample collection procedures such as phlebotomy and/or serum and plasma extraction. Here we trialled synthetic urine and RPMI media spiked with MR766 ZIKV culture as the simulated clinical matrix for our rapid Iso-ZIKV-Dx. Using the same spiked samples, we performed our Iso-ZIKV-Dx test concurrently with virus isolation and TaqMan RT-qPCR for a comprehensive comparison. The rapid Iso-ZIKV-Dx test exhibited a LOD of 3.56 × 10^7^ TCID_50_/mL of ZIKV in both synthetic urine (Figure 5) and RPMI media (Supplementary Figure 2). In RPMI culture media, this was quantified by TaqMan RT-qPCR to be equivalent to a Ct value of 35.91 and 449 copies/reaction. In urine, our LOD was quantified by TaqMan RT-qPCR to be equivalent to a Ct value of 37.71 and 34.28 copies/reaction.

## Discussion

Due to the lack of approved vaccines or specific therapeutics for ZIKV, rapid detection remains crucial in predicting and controlling potential outbreaks. ZIKV outbreaks often occur in rural and remote areas, making it necessary for ZIKV diagnostics to be suitable for use in resource-limited settings, whilst still meeting the requirement for sensitive and accurate detection of ZIKV infection. Isothermal NAAT techniques are a potential solution, especially when combined with safe and simple sample preparation methods, as they could be easily deployed in low-resource settings with minimal training requirements for healthcare workers. In this study, we evaluated a novel rapid ZIKV diagnostic (Iso-ZIKV-Dx) which combines a low-resource sample preparation, RT-RAA test, and LFD. We used a phylogenetically divergent and highly virulent strain (MR766) of ZIKV (Shao et al., 2017). In this study we demonstrated the analytical sensitivity of our RT-RAA test to be 500 copies/μL when using synthetic ZIKV RNA, and confirmed the test did not detect other co-circulating viruses. It should be noted that our synthetic ZIKV RNA generated from RNA transcripts of the NS1 gene whilst the co-circulating viruses consisted of total viral RNA. The rapid Iso-ZIKV-Dx detected 3.56 × 10^7^ TCID_50_/mL, equivalent to 34.28 copies/reaction RT-qPCR in synthetic urine spiked with ZIKV MR766 and offers improved safety for low-resource testing as the virus is inactivated in the very first step of the procedure. The novel sample preparation reagent, TNA-Cifer Reagent E, has previously been shown to inactivate other pathogens, such as DENV (Calvert et al., 2017), Nipah virus (Rossini et al., 2017) and Hendra virus (Jiangsu Qitian Gene Biotechnology Co., Ltd, 2023), ensuring operator safety in the event of other potential pathogens within the clinical sample. Our data emphasises the benefits of our uncomplicated sample preparation protocol, which proved effective in detecting ZIKV in urine samples. It is noteworthy that ZIKV RNA is known to have limited stability at room temperature, making it challenging to detect within a specific time frame (Tan et al., 2017). Of note, a comparative analysis on current RT-qPCR tests for ZIKV reported discrepancies in detection sensitivity and specificity amongst Asian versus African lineages (de Moraes et al., 2018) with particular emphasis on the difficulty observed among African lineages specifically. Therefore, the primers and probes used in this study were strategically designed to target highly conserved regions of the NS1 gene among both ZIKV lineages as illustrated via our bioinformatic alignment analysis of the most unique NS1 gene sequences.

RT-qPCR is still considered the most reliable technique for diagnosing ZIKV in clinical pathology, and is widely recognised as the gold standard method for detection. However, due to the RNA stability issues at room temperature reported in urine (Tan et al., 2017), methods such as RT-qPCR that require sample storage favour clinical matrices such as plasma, serum or whole blood. For comparison, in serum and plasma, ZIKV RT-qPCRs typically demonstrate detection limits between 1 and 64 copies/reaction (*n* = 31) (Pessôa et al., 2016). A comparative study of RT-qPCR versus RT-LAMP using urine to detect ZIKV, however, demonstrated a detection limit of 6 copies/reaction (*n* = 8) for RT-qPCR (Calvert et al., 2017). Our rapid Iso-ZIKV-Dx test showed similar levels of sensitivity, detecting as low as 34 copies/reaction in ZIKV-spiked synthetic urine. The Range of reported ZIKV concentration in urine was observed to be between 0.7 and 220^6^ copies/mL (Gourinat et al., 2015). The vastness of this range is thought to be due to the variability in ZIKV RNA stability, further supporting the need for a rapid sample processing method. Non-invasive clinical sample collections such as urine (Lamb et al., 2016) offer significant advantages for isothermal, POC compatible methods such as RT-RAA, RT-RPA, and RT-LAMP, due to the prolonged period of ZIKV RNA detectable throughout the infection period (Rossini et al., 2017). As such, our rapid Iso-ZIKV-Dx test could be valuable in overcoming the obstacle of testing urine samples by potentially detecting ZIKV directly at the point of sample collection. One study using RT-LAMP reported a LOD of 6.6 copies/reaction in urine (*n* = 63) (Castro et al., 2018), whilst another study achieved 6 copies/reaction in urine (*n* = 178) (Calvert et al., 2017). Although both RT-qPCR and RT-LAMP offer slightly improved sensitivity compared to the Iso-ZIKV-Dx test, their application as POC diagnostics in low-resource settings is limited. RT-qPCR requires highly skilled personnel, expensive machinery and laboratory-based sample preparation. While RT-LAMP is known for its simplicity, sensitivity and speed which resulted in wide scale application in low resource settings, it still needs heating machines capable of attaining temperatures of 55–65°C. In contrast, RT-RAA, similarly to RT-RPA can be performed at near-ambient temperature (37–40°C) and moreover has a higher tolerance to PCR inhibitors (Li et al., 2020). Both have previously required laboratory-based purification of RNA, whereas our Iso-ZIKV-Dx test does not require any sophisticated laboratory equipment, providing potential the entire procedure, from sample to result, be performed in low-resource near-patient settings. The ideal rapid POC test should provide a range of detection and diagnostic capabilities, as defined by the REASSURED criteria – Real-time connectivity, Ease of specimen collection, Affordable, Sensitive, Specific, User-friendly, Rapid, Equipment-free, and Deliverable to end-users (Land et al., 2019). Our data suggests that the Iso-ZIKV-Dx (sample preparation, RT-RAA, LFD) has potential to meet the REASSURED criteria, providing real-time connectivity through direct analogue reading of the result which could be performed at or near the patient in a low-resource clinic. Insofar as our assay has met pre-clinical and REASSURED criteria, our next phase of evaluation will involve clinical and in-field evaluation of the assay to evaluate clinical efficacy and performance among pathology of ZIKV infections. As our study only involved live MR766 strain of ZIKV, our hope would be to evaluate our ZIKV test with multiple strains within these clinical trials.

Due to manual steps in our assay, it should be acknowledged that screening of large-scale clinical samples would not occur as rapidly as standard RT-PCR assays. However, unlike RT-PCR, our rapid ZIKV test has been designed to be field deployable, requiring minimal resources and minimally trained staff. As a result, the time from sample preparation to diagnostic result is considerably faster than when compared to transportation and processing within central laboratory. Moreover, this particular attribute of our ZIKV test renders it a potential early warning screening tool in the face of a sudden outbreaks. A second potential challenge for our ZIKV test, and indeed many in-field nucleic acid amplification assays (Tang et al., 2016), is the risk of post amplification cross-contamination. A potential solution to this issue is the substitution of dTTP with dUTP in the amplification reactions. This approach has been shown to reduce cross-contamination in LAMP assays (Kil et al., 2015). However, to the authors knowledge, the feasibility of implementing such measure in recombinase-aided amplification assays has yet to be assessed. Therefore, a more feasible alternative is to utilise ‘U-Star’ disposable cartridges (TwistDx^™^, 2023b), which would reduce cross contamination occurrences by limiting external exposure of lateral flow strips to the environment.

Rapid detection of arboviruses such as ZIKV play a crucial role in limiting outbreaks that have already emerged or are currently on going. Additionally, surveillance and monitoring can serve to identify early signs of the pathogen and thereby prevent outbreaks through vector control. The study of vector competency and transovarial transmission of arboviruses such as ZIKV thus remain a particular area of focus. Due to factors such as climate change and increased urbanisation (Li et al., 2017) vector competency continues to broaden, further implicating the emergence of novel and existing viruses. A tool with in-field capabilities that provides robust and rapid results could be highly beneficial in epidemiology for monitoring ZIKV. To that end, further studies are warranted to determine if our Iso-ZIKV-Dx is compatible for detection of virus in mosquitoes, as was similarly performed for the TNA-Cifer Reagent E combined with RPA-LFD for detection of dengue virus in *Aedes aegpytii* mosquitoes. This would allow our Iso-ZIKV-Dx test to be extended beyond diagnostics alone, in potentially offering strategies toward aiding in surveillance and monitoring of viral vectors (Pollak et al., 2023b) and possibly reservoir hosts for arboviruses such as ZIKV.

## Conclusion

In conclusion, we developed a rapid, isothermal test for ZIKV that only requires incubation at 39°C and produces a real-time result in 30 min. Pre-clinical evaluation suggests that our Iso-ZIKV-Dx could offer promising innovations for diagnostics in ZIKV endemic areas. We demonstrate that our ZIKV test was able to detect as low as 500 copies/μL of synthetic ZIKV RNA and also, does not detect any other co-circulating arbovirus. We successfully established a simple sample preparation procedure which demonstrated that we were able to completely inactivate one of the highest virulent strains of ZIKV (MR766, African lineage) in 5 min at room temperature, whilst at the same time, extracting sufficient quantities of viral RNA for detection. By combining our simple sample preparation procedure with RT-RAA and Lateral Flow Strip detection, we successfully developed a robust rapid isothermal ZIKV diagnostic. Our data illustrated that our ZIKV test operates five times faster than ‘gold standard’ RT-qPCR and exhibited a detection limit of 34 RNA copies/reaction in spiked synthetic urine. Furthermore, due to the simplistic nature of our ZIKV test, we surmise that the applications of our Iso-ZIKV-Dx extend beyond detection alone, and could potentially provide innovations toward surveillance and monitoring for ZIKV and arboviruses alike.

## Data availability statement

The original contributions presented in the study are included in the article/Supplementary material, further inquiries can be directed to the corresponding authors.

## Author contributions

RB: conceptualization, data curation, formal analysis, investigation, methodology, resources, supervision, validation, visualization, writing – original draft, and writing – review and editing. NP, JM, and DM: conceptualization, formal analysis, funding acquisition, methodology, project administration, resources, supervision, and writing – review and editing. JH-P: resources and writing – review and editing. All authors contributed to the article and approved the submitted version.
