# Supplementary material for: Development and pre-clinical evaluation of a Zika virus diagnostic for low resource settings
Source: Front Microbiol. 2023 Nov 20;14:1214148. doi: 10.3389/fmicb.2023.1214148 (PMC10694267; doi:10.3389/fmicb.2023.1214148)
Supplement: Supplementary file 1 [file Data_Sheet_1.docx]

# Supplementary Information


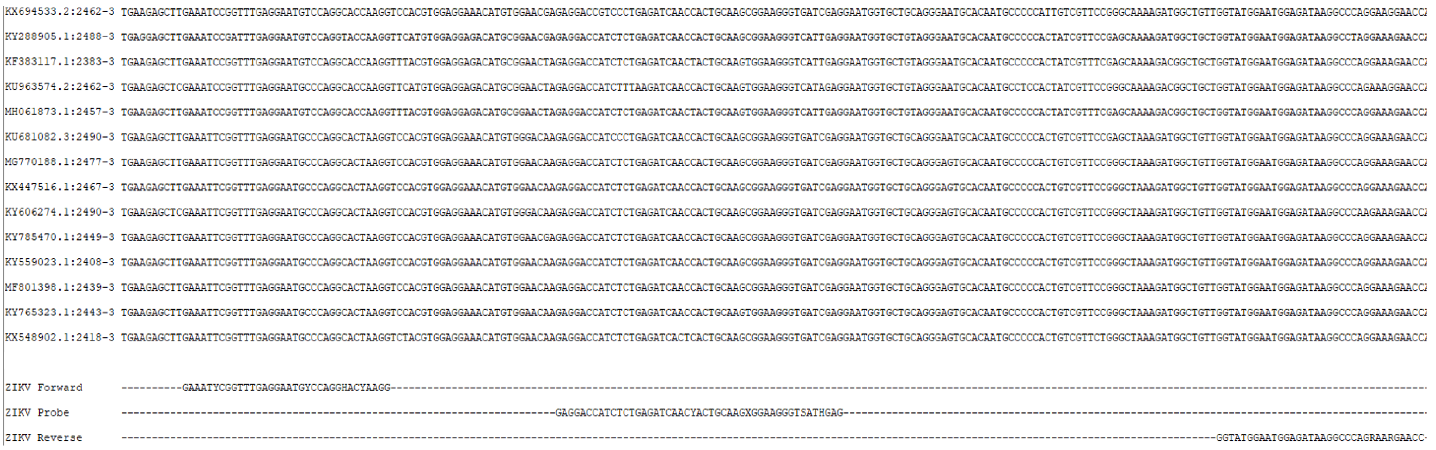


**Figure 1. Multiple sequence alignment of ZIKV NS1 gene showing 14 most unique (African and Asian lineages) regions targeted by RT-RAA forward primer, reverse primer and probe.**

KX694533.2:2462-3517 Zika virus strain ZIKV/Aedes aegypti/MYS/P6-740/1966, complete genome, KY288905.1:2488-3542 Zika virus strain MP1751, complete genome, KF383117.1:2383-3437 Zika virus strain ArD128000 polyprotein gene, complete cds, KU963574.2:2462-3517 Zika virus isolate ZIKV/Homo sapiens/NGA/IbH-30656_SM21V1-V3/1968 polyprotein (GP1) gene, complete cds, MH061873.1:2457-3511 Zika virus strain ZIKV/Macaca mulatta/UGA/MR-766-VEROE6-FA12-P7_01/1947, complete genome, KU681082.3:2490-3545 Zika virus isolate Zika virus/H.sapiens-tc/PHL/2012/CPC-0740, complete genome, MG770188.1:2477-3532 Zika virus isolate SV0127-14, complete genome, KX447516.1:2467-3522 Zika virus isolate 1_0111_PF polyprotein gene, complete cds, KY606274.1:2490-3545 Zika virus isolate mex39/Mexico/2016 polyprotein gene, complete cds, KY785470.1:2449-3504 Zika virus isolate Zika virus/H.sapiens-wt/DOM/2016/MA-WGS16-014-SER polyprotein gene, complete cds, KY559023.1:2408-3463 Zika virus isolate ZIKV/Homo_sapiens/Brazil/2016/ZBRX130 polyprotein gene, complete cds, MF801398.1:2439-3494 Zika virus isolate ZIKV/Homo sapiens/MEX/2016/mex17 polyprotein gene, complete cds, KY765323.1:2443-3498 Zika virus strain ZIKV/Homo sapiens/NIC/6188_13A1/2016, complete genome, KX548902.1:2418-3473 Zika virus isolate ZIKV/COL/FCC00093/2015 polyprotein gene, complete cds, KX548902.1:2418-3473 Zika virus isolate ZIKV/COL/FCC00093/2015 polyprotein gene, complete cds. ‘X’ indicates position of internal dS spacer on the probe.


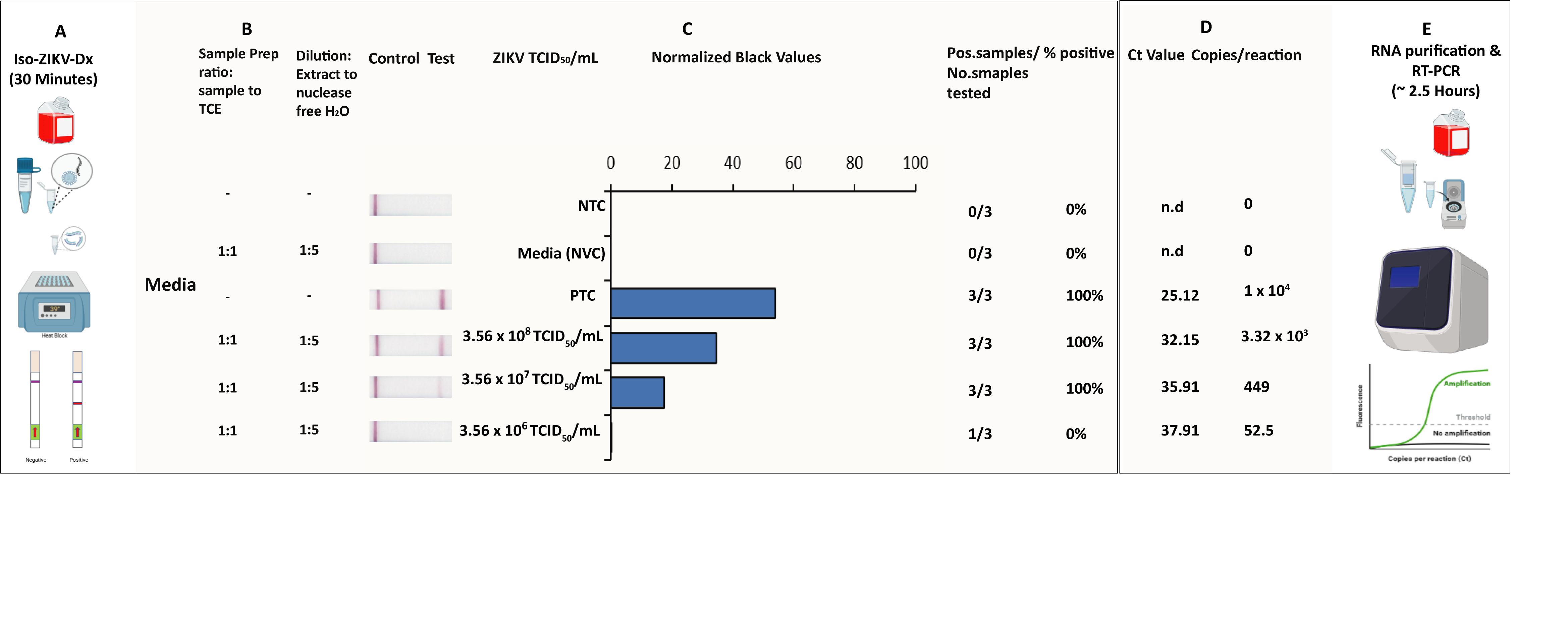


Figure 2. **Rapid Iso-ZIKV-Dx of RPMI media samples spiked with ZIKV (MR766).** A) Workflow, equipment needed and time frame of Iso-ZIKV-Dx. B) Sample processing conditions including sample to reagent ratio and processed sample dilution ratio. C) Sample description and quantities (NTC, non-template control; PTC, positive template control, synthetic ZIKV RNA transcripts 10^6^ copies/µl); NVC, no virus control, RPMI media. Scanned lateral flow strips showing test and control bands observable by naked eye. Normalised pixel densities (black values) from the displayed lateral flow strips. D) Comparative Ct values and copies/reaction quantified via TaqMan RT-qPCR .E) Workflow, equipment and time frame involved in conventional ZIKV RT-qPCR diagnostic.
